# Total laparoscopic excision of pelvic retroperitoneal ganglioneuroma: a case report and review of the literature

**DOI:** 10.3389/fsurg.2025.1707642

**Published:** 2026-01-15

**Authors:** Kangyun Lan, Hongfan Ding, Wenli Liu, Hanmei Tang, Guangyuan Chen, Ping Huang

**Affiliations:** Department of Obstetrics and Gynecology, Shenzhen Bao'an District Songgang People’s Hospital, Shenzhen, Guangdong, China

**Keywords:** case report, laparoscopic surgery, pediatric surgery, pelvic ganglioneuroma, retroperitoneal tumor

## Abstract

Ganglioneuroma is defined as a rare, benign neurogenic tumor. This paper reports a case of pelvic retroperitoneal ganglioneuroma in a 12-year-old girl and reviews the relevant literature. The patient was found to have a pelvic retroperitoneal mass incidentally during a health check-up and had no significant clinical symptoms. Gynecological ultrasound revealed a hypoechoic mass posterior to the uterus, measuring approximately 63 × 52 mm, with clear boundaries. No obvious abnormalities were observed in the uterus and adnexa. Pelvic CT showed a well-defined, low-density mass measuring 59 mm × 55 mm with mild enhancement at the posterior margin, the adjacent coccygeal bone showed discontinuity and was closely related to the mass, the rectum was displaced anteriorly with clear demarcation from the mass, and no enlarged lymph nodes or effusion were found in the pelvis. The tumor was successfully and completely resected via laparoscopy, and pathological examination confirmed the diagnosis of ganglioneuroma. The standard surgical methods and approaches for retroperitoneal ganglioneuroma have not yet been established. The successful diagnosis and treatment of this case highlight the importance of accurate preoperative assessment and appropriate surgical planning. The literature review summarizes the clinical manifestations, diagnostic methods, and therapeutic strategies of ganglioneuroma, aiming to provide references for clinicians.

## Introduction

Ganglioneuroma is most commonly found in the posterior mediastinum and retroperitoneum (1). Retroperitoneal ganglioneuroma is a primitive, rare benign tumor arising from sympathetic nerve differentiation, composed of mature Schwann cells, ganglion cells, and nerve fibers, without cellular atypia, and showing no mitotic activity or necrosis (2). Retroperitoneal ganglioneuromas are typically nonfunctional and asymptomatic (3) until they reach a large size, causing symptoms due to local expansion and compression of adjacent structures, such as back pain, abdominal pain, vomiting, constipation, weight loss, and urinary symptoms (1). In some cases, metabolic activity may lead to the secretion of catecholamines, vasoactive intestinal peptide, or androgens, resulting in symptoms such as hypertension, diarrhea, and virilization. During surgery, these catecholamines may trigger hypertensive crises (4, 5). This article reports a case of a patient who underwent laparoscopic resection of a pelvic retroperitoneal ganglioneuroma, providing a reference for clinical practice.

### Case presentation

**Figure 1:**
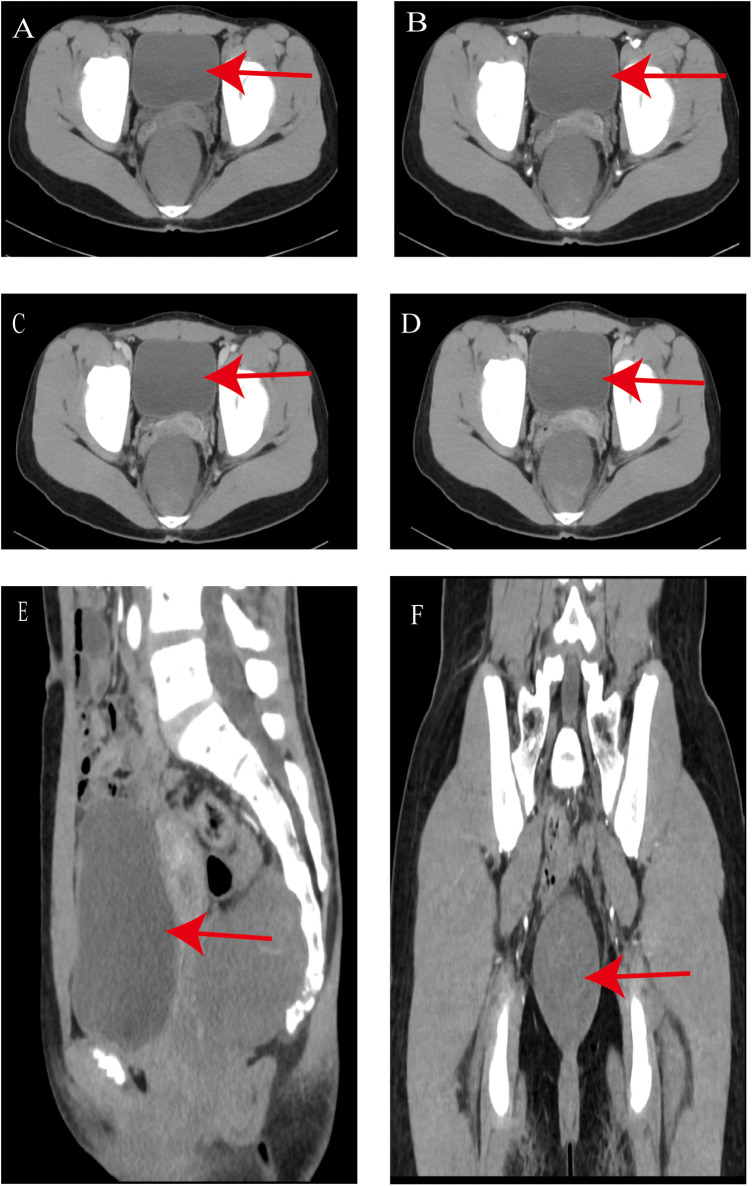
Preoperative CT findings (May 25, 2022) **(A)** Non-contrast (CT value approx. 27 HU); **(B)** Arterial phase (CT value approx. 30 HU); **(C)** Venous phase (CT value approx. 32 HU); **(D)** Delayed phase (CT value approx. 35 HU) Mild enhancement is observed; **(E)** Sagittal view; **(F)** Coronal view.

**Figure 2:**
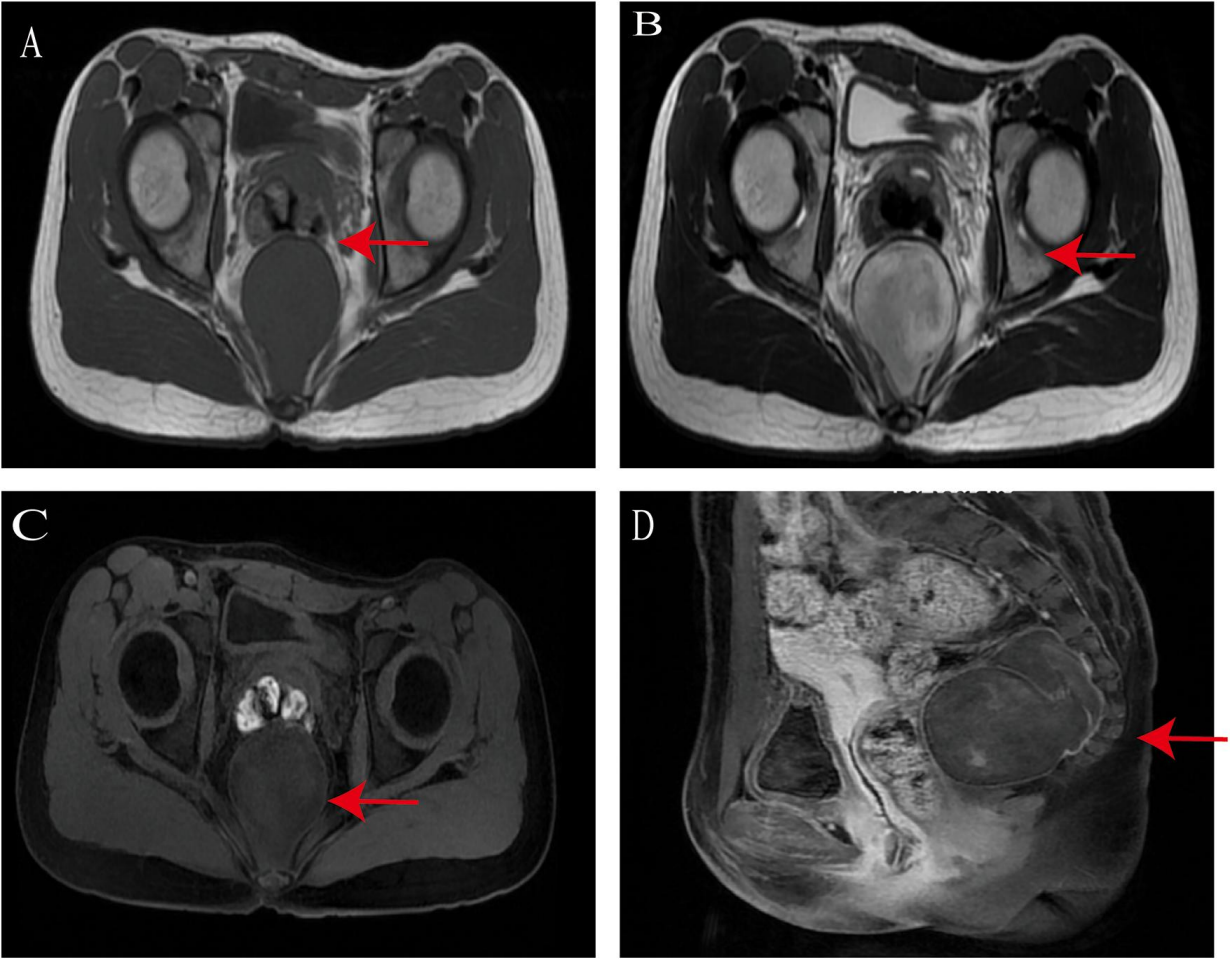
Preoperative MRI findings (May 22, 2022) **(A)** T1: Low signal intensity; **(B)** T2: high signal intensity with heterogeneous intensity; **(C)** T1 FSE; **(D)** contrast-enhanced scan shows mild heterogeneous enhancement compared to T1 FSE.

On May 24, 2022, a 12-year-old female patient, was admitted for further evaluation and management of a pelvic mass that had been detected on ultrasound 6 days before. Laboratory tests upon admission showed CA199: 43.46 U/mL, CA125: 20.57 U/mL, HE4: 32.79 U/mL, with no significant abnormalities in AFP or CEA. Gynecological ultrasound revealed a hypoechoic occupying lesion posterior to the uterus (retroperitoneal solid mass could not be excluded), measuring approximately 63 × 52 mm, oval-shaped, with heterogeneous internal echogenicity and clear boundaries, the uterus and adnexa showed no significant abnormalities. Pelvic spiral non-contrast and contrast-enhanced CT (Figure 1) indicated a retroperitoneal presacrococcygeal occupying lesion (59 × 55 mm, with smooth and clear margins), suggestive of a neurogenic tumor, although other possibilities could not be entirely ruled out; a few strip-like areas of significant enhancement were observed at the posterior edge of the lesion, with relative discontinuity of the adjacent coccygeal bone showing close relationship to the lesion; the rectum was displaced anteriorly but clearly demarcated from the lesion; no definite enlarged lymph nodes or fluid collection were seen in the pelvis. Pelvic MRI (Figure 2) confirmed a retroperitoneal presacrococcygeal occupying lesion (52 × 49 mm, likely benign neurogenic tumor?); the rectum was anteriorly displaced with clear demarcation; no definite enlarged lymph nodes were found in the pelvis. High-resolution chest CT, abdominal ultrasound of the liver, gallbladder, pancreas, and spleen, and urinary system ultrasound all showed no significant abnormalities. The patient underwent laparoscopic surgical resection of a pelvic retroperitoneal tumor via a transperitoneal approach on May 28, 2022.

The surgical procedure is shown in the video (Supplementary Video S1). Following the establishment of the transabdominal laparoscopic approach, a well-defined mass measuring approximately 6 × 6 × 5 cm^3^ was observed in the retroperitoneal pelvic cavity, anterior to the sacrococcyx and posterolateral to the rectum. The mass had a smooth surface, was firm in consistency, and exhibited poor mobility. Given the patient's nulliparous status, which precluded transvaginal uterine manipulation, the uterine fundus was suspended using a figure-of-eight suture, and a needle holder was introduced through a suprapubic auxiliary port to elevate the uterus and improve exposure. Using an ultrasonic scalpel, the posterior peritoneum was incised along the left side of the rectum starting at the level of the third sacral vertebra, opening the rectovaginal space. The rectum was mobilized and retracted to the right. Simultaneously, the assistant performed a digital rectal examination, palpated the mass, and gently pushed it upward to further improve exposure of the tumor site. Subsequently, the ultrasonic scalpel was used to dissect along the tumor capsule in a superior-to-inferior and medial-to-lateral direction, sequentially separating the planes between the tumor and the rectum, sacrococcyx, and lateral pelvic wall, until the tumor was fully exposed. The tumor was noted to have an intact capsule, clear boundaries from the surrounding tissues, and a hard consistency. A distinct vascular pedicle, surrounded by multiple branching and interconnecting vessels, was observed at its site of attachment to the sacrum. The tumor's blood supply was progressively coagulated and transected using the ultrasonic scalpel and a vessel-sealing system. Finally, the tumor was completely enucleated.

**Figure 3:**
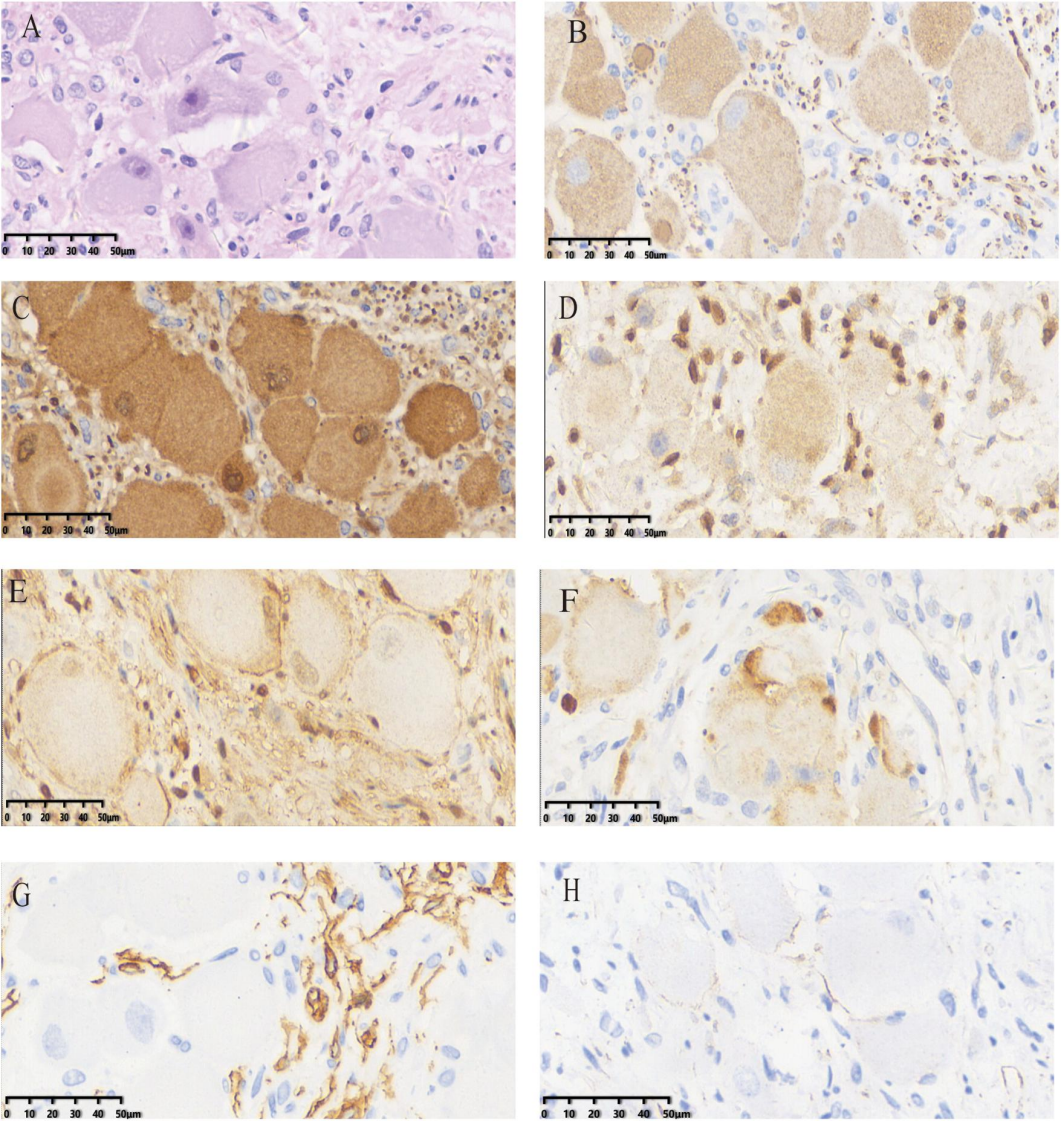
Histopathological examination and immunohistochemical analysis of the patient **(A)** HE staining; immunohistochemical analysis: **(B)** NF positive; **(C)** NSE positive; **(D)** SOX10 positive; **(E)** S100 positive; **(F)** Syn positive; **(G)** CD34 negative; **(H)** GFAP negative.

**Figure 4:**
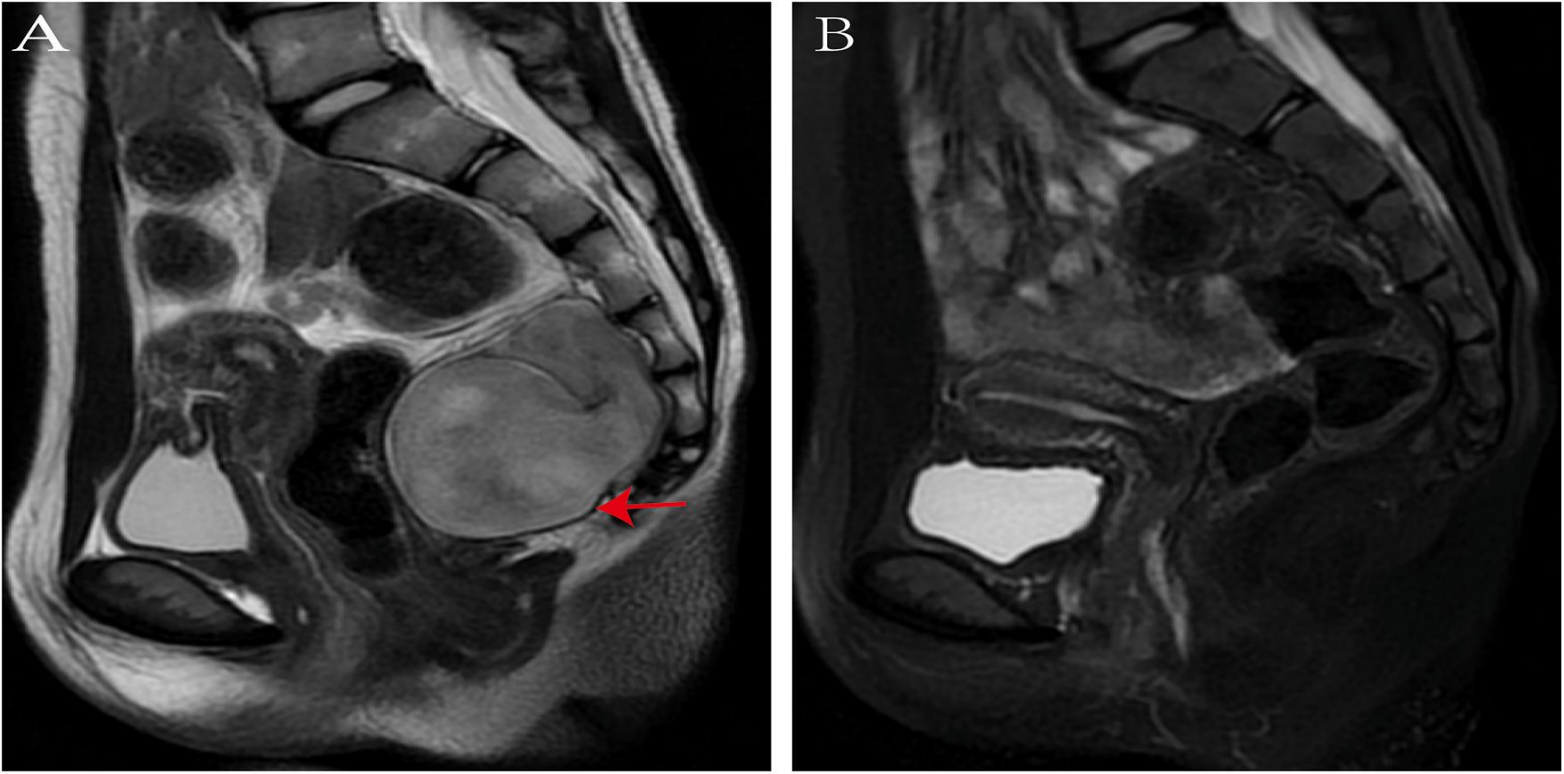
Preoperative and postoperative MRI findings **(A)** preoperative MRI; **(B)** postoperative MRI.

Postoperative pathological examination (Figure 3) confirmed ganglioneuroma. Immunohistochemistry results were positive for NF, NSE, SOX10, S100, and SYN, and negative for GFAP and CD34. The patient recovered well after surgery. Follow-up MRI (Figure 4) performed postoperatively showed no obvious signs of tumor recurrence or residual lesion in the surgical area after resection of the original retroperitoneal presacrococcygeal occupying lesion. A small amount of pelvic fluid collection was noted. The patient was advised to continue follow-up monitoring. This study was approved by the Medical Ethics Committee of Songgang People's Hospital of Bao'an District, Shenzhen (Approval No. IRB-PJ-2025-109).

## Discussion

The compressive effects of retroperitoneal ganglioneuroma on the pancreas, duodenum, and right kidney, as well as displacement of the inferior vena cava, can lead to chronic, progressive, and intractable abdominal pain (6). Anorexia and vomiting may also occur (7), along with leg pain due to nerve compression (8, 9). Retroperitoneal ganglioneuromas can arise anywhere along the paravertebral sympathetic plexus and occasionally in the adrenal medulla. As the tumor enlarges, it may compress adjacent organs or structures, manifesting as lower back pain (8, 10) or persistent back pain radiating from the thoracic region to the right abdomen (11). Large lumbar ganglioneuromas may present with low back pain radiating to the right lower limb (12). Left adrenal ganglioneuromas can cause hypertension (13, 14) and must be differentiated from pheochromocytom (14). In this case, the patient did not exhibit symptoms such as abdominal pain, hypertension, anorexia, or vomiting, highlighting the importance of routine health examinations in the general population for early detection and intervention of retroperitoneal ganglioneuromas.

Retroperitoneal ganglioneuromas commonly occur in the adrenal region (13), adjacent to the portal vein, inferior to the liver and to the right of the inferior vena cava, near the inferior mesenteric artery and left common iliac artery (15), in the parapharyngeal space (16, 17), and around the renal area (6). Spinal ganglioneuromas predominantly affect females (81%) and can be found in the cervical (9.5%), cervicothoracic (4.76%), thoracolumbar (19.04%), lumbar (19.04%), and sacral regions (4.76%) (18). The tumor in this case was located in the pelvic cavity without involvement of critical organs or blood vessels, providing diagnostic insight for clinicians evaluating pelvic masses as potential retroperitoneal ganglioneuromas and emphasizing the need to assess adjacent anatomical structures.

Retroperitoneal ganglioneuromas often exhibit a characteristic pattern of encasing blood vessels (2), such as the abdominal aorta, inferior vena cava, and inferior mesenteric artery. For instance, a large right subhepatic retroperitoneal ganglioneuroma in a 33-year-old female was reported to slide between and encase the inferior vena cava and aorta, displacing the main portal vein anteriorly and wrapping around the celiac trunk, aorta, and inferior vena cava by more than 180°. The tumor also encased the origin of the right renal artery and contacted the right renal vein (19). Other studies have noted that resection of retroperitoneal ganglioneuromas may expose the inferior vena cava, aorta, right renal artery, left renal vein, and left superior renal vein (20). Peripancreatic retroperitoneal ganglioneuromas can involve dual vascular structures (celiac trunk and superior mesenteric artery) (21). Multifocal clustered ganglioneuromas may infiltrate retroperitoneal fat, become inseparable from the inferior vena cava, and displace it anteriorly (17). In this case, the tumor did not encase major vessels, as preoperatively assessed by CT and confirmed intraoperatively. This underscores the importance of preoperative evaluation and intraoperative vigilance regarding vascular involvement to avoid excessive bleeding.

Imaging features of retroperitoneal ganglioneuromas typically include hypoechoic lesions with moderate blood flow signals on ultrasound (6), and soft-tissue density lesions with mild to moderate enhancement on CT (6). Due to abundant stromal tissue and predominant mucoid matrix, contrast agents gradually accumulate in the extracellular space and clear slowly, resulting in progressive enhancement during arterial, venous, and delayed phases of contrast-enhanced CT (22). In this patient, ultrasound revealed a hypoechoic mass, and 64-slice spiral CT showed attenuation values of approximately 27 HU on non-contrast images, with mild enhancement reaching 30 HU, 32 HU, and 35 HU in the arterial, venous, and delayed phases, respectively. These findings supported the preoperative diagnosis of retroperitoneal ganglioneuroma. On MRI, most retroperitoneal ganglioneuromas exhibit a visible capsule, homogeneous or heterogeneous low signal intensity on T1-weighted imaging (T1WI), and heterogeneous high signal intensity on T2-weighted imaging (T2WI) (6, 23). Many patients also demonstrate a “whorled sign” on MRI (23). In this case, the MRI findings included low T1WI and high T2WI signals.

Diagnostically, one reported case of a large retroperitoneal tumor was initially misdiagnosed as liposarcoma due to similar enhancement patterns on contrast-enhanced CT and the detection of adipocytes via cytological techniques in ganglioneuroma, highlighting the importance of differentiating ganglioneuroma from liposarcoma (24). Large retroperitoneal ganglioneuromas are often misdiagnosed as mesenteric cysts, and preoperative histopathological confirmation is recommended (25). Multimodal ultrasound imaging with ultrasound-guided core needle biopsy can facilitate preoperative diagnosis of retroperitoneal ganglioneuroma (26).

A study reported 30 cases of retroperitoneal ganglioneuroma resected laparoscopically, all completed successfully. The lesions included two adrenal cysts and 26 adrenal tumors (23 suspected ganglioneuromas), one left para-aortic ganglioneuroma, and one right retrocaval ganglioneuroma. Among these, 24 were resected via a retroperitoneal approach and six via a transperitoneal approach (27). Another case involved an open resection of a right retroperitoneal ganglioneuroma at the L5-S1 level adjacent to the iliac vessels and right ureter, with bilateral ureteral stenting (28). Robot-assisted resection of preperitoneal/retroperitoneal masses and attempted total resection of retroperitoneal ganglioneuroma via a posterior laminectomy/microsurgical approach have been described for tumors centered at the right D12-L1 neural foramen with intraspinal extension, compressing and laterally displacing the dural sac, and extending paravertebrally between D11 and L2 (11). Intraoperative blood loss ≥400 mL has been associated with maximal tumor size on axial and coronal imaging, encasement of unilateral or bilateral renal pedicles, encasement of the aorta and/or inferior vena cava, and the presence of a whorled sign on MRI (29). When a retroperitoneal ganglioneuroma compresses the inferior vena cava, a guidewire can be inserted from the right femoral vein into the inferior vena cava during laparoscopic resection to facilitate emergency balloon insertion in case of hemorrhage (30). Large lumbar ganglioneuromas are typically resected via a retroperitoneal approach (12).

In this study, preoperative imaging (CT and MRI) indicated that the tumor was located in the pelvis, within the retroperitoneum, anterior to the sacrococcygeal vertebrae, and at the level of the upper rectum. Digital rectal examination allowed palpation of the inferior pole of the mass approximately 3–4 cm above the anal verge. A retroperitoneal approach would have carried risks such as injury to the sacrococcygeal bone, significant intraoperative bleeding, and conspicuous surgical scarring. Given that the patient was an adolescent female, prominent scarring could have adversely affected her long-term psychological well-being and self-confidence. A minimally invasive approach not only better aligns with her aesthetic needs but is also consistent with current trends in surgical management. Conversely, a traditional open laparotomy—due to the deep-seated location of the tumor and limited exposure—could have resulted in incomplete resection and would similarly have left a larger scar.

After comprehensive evaluation, we determined that the transabdominal laparoscopic approach offered advantages such as greater depth of field and a wider field of view. These characteristics facilitate clear exposure of the tumor within the deep pelvic structures, enable complete resection, and help minimize intraoperative blood loss. Therefore, the decision was made to proceed with transabdominal laparoscopic resection.

Intraoperatively, considering the patient had no history of sexual activity, vaginal uterine manipulation was not feasible. Instead, a “figure-of-eight” suture was placed at the uterine fundus to secure and elevate the uterus, and a needle holder was introduced through a suprapubic auxiliary port to further improve exposure. Additionally, given the firm, leiomyoma-like consistency of the tumor, a myoma screw was used to optimize traction and exposure. These two techniques contributed to the successful complete resection of the mass, with an estimated blood loss of 100 mL and no injury to surrounding structures. This approach may serve as a useful reference for other surgeons.

A study of 32 patients with retroperitoneal ganglioneuroma reported residual tumor in four cases after surgery, none of which progressed or caused symptoms. No recurrences were observed during follow-up (6). A multi-institutional study of 328 patients with retroperitoneal ganglioneuroma found 92.2% remained stable in size, among tumors under surveillance (median follow-up 1.9 years). For resected tumors (median follow-up 3.0 years), 84.4% were disease-free, while four patients (1.9%) experienced recurrence (31). In this case, the patient underwent complete surgical resection, and histopathology confirmed ganglioneuroma. No recurrence has been detected during regular follow-up to date.

## Conclusion

In summary, this case demonstrates that total laparoscopic excision is a feasible and safe approach for pelvic retroperitoneal ganglioneuromas without major vascular involvement or critical organ adjacency. Preoperative imaging, particularly CT and MRI, plays a crucial role in surgical planning by accurately delineating tumor characteristics and anatomical relationships. Complete resection remains the cornerstone of treatment, ensuring excellent long-term outcomes with minimal risk of recurrence. This report further highlights the importance of routine health examinations for early detection and intervention of asymptomatic retroperitoneal tumors.

## Data Availability

The original contributions presented in the study are included in the article/Supplementary Material, further inquiries can be directed to the corresponding authors.

## Supplementary material

Supplementary Video S1 Surgical video.

## References

[1] Wang X Yang L Shi M Liu X Liu Y Wang J. Retroperitoneal ganglioneuroma combined with scoliosis: a case report and literature review. Medicine (Baltimore). (2018) 97(37):e12328. 10.1097/MD.0000000000012328 30212980 PMC6156057

[2] Jing WS Yu QS Pan JF Wang Z. Retroperitoneal ganglioneuroma: a case report. Chin J Surg Integr Tradit West Med. (2014) 20(5):553–4. 10.3969/j.issn.1007-6948.2014.05.036

[3] Adas M Koc B Adas G Ozulker F Aydin T. Ganglioneuroma presenting as an adrenal incidentaloma: a case report. J Med Case Rep. (2014) 8:131. 10.1186/1752-1947-8-131 24779851 PMC4031973

[4] Moriwaki Y Miyake M Yamamoto T Tsuchida T Takahashi S Hada T Retroperitoneal ganglioneuroma: a case report and review of the Japanese literature. Intern Med. (1992) 31(1):82–5. 10.2169/internalmedicine.31.82 1568049

[5] Radin R David CL Goldfarb H Francis IR. Adrenal and extra-adrenal retroperitoneal ganglioneuroma: imaging findings in 13 adults. Radiology. (1997) 202(3):703–7. 10.1148/radiology.202.3.9051020 9051020

[6] Xiao J Zhao Z Li B Zhang T. Primary retroperitoneal ganglioneuroma: a retrospective cohort study of 32 patients. Front Surg. (2021) 8:642451. 10.3389/fsurg.2021.642451 34095202 PMC8176303

[7] Kitazawa M Matsuhashi N Imai T Iwata Y Takahashi T Yoshida K. Total laparoscopic excision of retroperitoneal ganglioneuroma: a case report. Int J Surg Case Rep. (2021) 83:106053. 10.1016/j.ijscr.2021.106053 34098185 PMC8187827

[8] Papaetis GS Georgiadis CP Tsitskari MA Constantinou PG Antoniou AP. Retroperitoneal ganglioneuroma causing chronic lower back and leg pain in an 80-year-old man: a case report. Ann Med Surg (Lond). (2021) 61:101–3. 10.1016/j.amsu.2020.12.027 33437470 PMC7785993

[9] Saadi A Chebbi S Mokadem S Kacem LBH Chakroun M Slama MRB. Retroperitoneal ganglioneuroma: a five-case series from a single Tunisian center. Int J Surg Case Rep. (2023) 111:108840. 10.1016/j.ijscr.2023.108840 37734125 PMC10518479

[10] Kordeni K Chardalias L Pantiora E Massaras D Papadopoulos K Primetis E Retroperitoneal ganglioneuroma presenting as lower back pain. J Surg Case Rep. (2022) 2022(4):rjac082. 10.1093/jscr/rjac082 35444793 PMC9015710

[11] Tavares WM de Franca SA Vasconcelos AS Parra DSL Araújo SRR Teixeira MJ. Robotic and standard surgical intervention as adjunct therapies for retroperitoneal ganglioneuroma resection: a case report. BMC Surg. (2021) 21(1):143. 10.1186/s12893-021-01146-x 33740932 PMC7980646

[12] Altalhi L Alayyaf A Bin-Mahfooz M Alhumoudi D Alkhaibary A AlSufiani F Giant ganglioneuroma of the lumbar spine: a rare cause of radiculopathy. Case Rep Surg. (2024) 2024:9477892. 10.1155/2024/9477892 38883268 PMC11178425

[13] Aynaou H Salhi H El Ouahabi H. Adrenal ganglioneuroma: a case report. Cureus. (2022) 14(8):e27634. 10.7759/cureus.27634 36072207 PMC9438294

[14] Eldin MM Daum RE Kumar P Uecker J. Adrenal ganglioneuroma: diagnosis, presentation, and management of a rare tumor. Cureus. (2023) 15(6):e39977. 10.7759/cureus.39977 37415991 PMC10321198

[15] Yang WB Cao K Zhang B Wen Q Wang RH. Clinical analysis on 18 cases of retroperitoneal ganglioneuroma. Chin J Bases Clin Gen Surg. (2017) 24(9):1100–5. 10.7507/1007-9424.201703126

[16] Li F Feng H Liao J Bao Y Xu S Qin G. Parapharyngeal space ganglioneuroma: clinical experience and review of the literature. Ear Nose Throat J. (2023) 102(12):765–71. 10.1177/01455613221142658 36450599

[17] Rich JM Duddalwar VA Aron M Ter-Oganesyan R Hu P Chopra S Localized multifocal retroperitoneal ganglioneuroma with an infiltrative appearance on imaging: a case report. Case Rep Oncol. (2023) 16(1):1142–7. 10.1159/000534060 37900859 PMC10601809

[18] Bteich F Larmure O Stella I Klein O Joud A. Spinal ganglioneuroma: a rare and challenging tumor in the pediatric population. Childs Nerv Syst. (2024) 40(12):4301–7. 10.1007/s00381-024-06603-5 39243334

[19] Bourouail O Kada A Bahou K Sekkat H Zouaidia F Derquaoui S Large retroperitoneal ganglioneuroma revealed by a left ovarian endometrioma: a case report. SAGE Open Med Case Rep. (2024) 12:2050313(241252744. 10.1177/2050313X241252744 PMC11097720 38756330

[20] Sowa A Holecki M Mrowiec S Migacz M Śnietura M Jabłońska B. Asymptomatic retroperitoneal ganglioneuroma adjacent to the abdominal organs and large abdominal vessels. Pol Arch Intern Med. (2025) 135(2):16893. 10.20452/pamw.16893 39688517

[21] Avila-Sanchez P Barron-Cervantes NM Martinez-Esteban A Chan-Nuñez LC. Retroperitoneal peripancreatic ganglioneuroma encasing the celiac trunk and superior mesenteric artery. Cureus. (2024) 16(1):e52405. 10.7759/cureus.52405 38371023 PMC10869318

[22] Li YJ Fu DL Zhang X Guo Y Sun X Luo CH. Preoperative assessment and diagnosis of CT in complicated retroperitoneal ganglioneuroma. Chin J Med Imaging. (2023) 31(7):747–50. 10.3969/j.issn.1005-5185.2023.07.014

[23] Pacella G Brunese MC Donnarumma F Barrassi M Bellifemine F Sciaudone G. Imaging of ganglioneuroma: a literature review and a rare case of cystic presentation in an adolescent girl. Diagnostics (Basel). (2023) 13:13. 10.3390/diagnostics13132190 PMC10341194 37443583

[24] Bapir R Hawrami T Aghaways I Ali R Hiwa D Hussein D A huge retroperitoneal ganglioneuroma in a middle-aged patient: report of a diagnostically challenging case with review of the literature. Oncol Lett. (2022) 24(6):449. 10.3892/ol.2022.13569 36420079 PMC9647783

[25] Shah D Chaudhary SR Khan S Mallik S. Overreliance on radiological findings leading to misdiagnosed giant retroperitoneal ganglioneuroma: a case report and literature review. Cureus. (2023) 15(8):e43914. 10.7759/cureus.43914 37746449 PMC10512760

[26] Feng L Wang Y. A case report of multimodal ultrasound imaging in the diagnosis of giant retroperitoneal ganglioneuroma. Cancer Innov. (2023) 2(5):433–7. 10.1002/cai2.73 38090383 PMC10686167

[27] Bin Gheshayan S Alsadun D Alharbi A Alselaim NA. Laparoscopic resection of retroperitoneal ganglioneuroma (report of 30 cases). Chin J Urol. (2008) 29(3):188–91. 10.3321/j.issn:1000-6702.2008.03.011

[28] Bin Gheshayan S Alsadun D Alharbi A Alselaim N. A large retroperitoneal ganglioneuroma presenting with an abdominal pain: a case report. Cureus. (2021) 13(12):e20600. 10.7759/cureus.20600 35103176 PMC8778648

[29] Zhang Q-W Song T Yang P-P Hao Q. Retroperitoneum ganglioneuroma: imaging features and surgical outcomes of 35 cases at a Chinese institution. BMC Med Imaging. (2021) 21(1):114. 10.1186/s12880-021-00643-y 34294064 PMC8296746

[30] Sugai Y Yamoto M Obayashi J Tsukui T Nomura A Miyake H Laparoscopic resection for retroperitoneum ganglioneuroma with supine hypotension syndrome. Surg Case Rep. (2024) 10(1):192. 10.1186/s40792-024-01992-w 39160326 PMC11333399

[31] Noh S Nessim C Keung EZ Roland CL Strauss D Sivarajah G Retrospective analysis of retroperitoneal-abdominal-pelvic ganglioneuromas: an international study by the transatlantic australasian retroperitoneal sarcoma working group (TARPSWG). Ann Surg. (2023) 278(2):267–73. 10.1097/SLA.0000000000005625 35866666 PMC10191524

